# Metabolite Profiling and Antimicrobial Activities of *Brassica rapa* ssp. *narinosa* (Tatsoi), *Brassica rapa* var. *narinosa* × *chinensis* (Dacheongchae), and *Brassica rapa* ssp. *chinensis* (Pakchoi)

**DOI:** 10.3390/molecules30081693

**Published:** 2025-04-10

**Authors:** Chang-Ha Park, Hyeon-Ji Yeo, Young-Jin Park, Haejin Kwon, Jongki Cho, Sun-Ok Chung, Geung-Joo Lee, Jae-Kwang Kim, Sang-Un Park

**Affiliations:** 1 Department of Smart Farm, Namseoul University, 91 Daehak-ro, Cheonan-si 31020, Republic of Korea; parkch@nsu.ac.kr; 2 Department of Crop Science, Chungnam National University, 99 Daehak-ro, Daejeon 34134, Republic of Korea; guswl7627@gmail.com; 3 Division of Life Sciences and Convergence Research Center for Insect Vectors, Incheon National University, Incheon 22012, Republic of Korea; pyj2050@inu.ac.kr; 4 Department of Smart Agriculture Systems, Chungnam National University, 99 Daehak-ro, Daejeon 34134, Republic of Korea; kwonhaejin42@o.cnu.ac.kr (H.K.); sochung@cnu.ac.kr (S.-O.C.); gjlee@cnu.ac.kr (G.-J.L.); 5 College of Veterinary Medicine, Chungnam National University, 99 Daehak-ro, Daejeon 34134, Republic of Korea; cjki@cnu.ac.kr; 6 College of Veterinary Medicine and Research Institute for Veterinary Science, Seoul National University, 1 Gwanak-ro, Seoul 08826, Republic of Korea; 7 Department of Agricultural Machinery Engineering, Graduate School, Chungnam National University, 99 Daehak-ro, Daejeon 34134, Republic of Korea; 8 Department of Horticulture, Chungnam National University, 99 Daehak-ro, Daejeon 34134, Republic of Korea

**Keywords:** *Brassica rapa* cultivars, Tatsoi, Dacheongchae, Pakchoi, antibacterial activity against multidrug-resistant pathogens, metabolic profile

## Abstract

Pakchoi and Tatsoi are agriculturally and commercially important subspecies of *Brassica rapa*. Dacheongchae is a new crop generated via the hybridization of Tatsoi and Pakchoi. Metabolite profiles of carbohydrates, sugar alcohols, amines, amino acids, carotenoids, phenolics, organic acids, and glucosinolates were carried out in the three *B. rapa* cultivars. The majority of amino acids were higher in Dacheongchae than in Pakchoi and Tatsoi. In addition to the amino acid content, higher contents of phenolic compounds and carotenoids were obtained in Dacheongchae. Similarly, Dacheongchae and Pakchoi contained higher amounts of glucosinolates compared with Tatsoi. Pakchoi, Tatsoi, and Dacheongchae showed marked antimicrobial activity against *Bacillus cereus*, *Escherichia coli*, *Candida albicans*, *Pseudomonas aeruginosa*, *Proteus mirabilis*, and methicillin-resistant *P. aeruginosa*. Furthermore, Dacheongchae extracts exhibited only the inhibition activity of *Salmonella paratyphi*. Consistent with these higher amounts of bioactive compounds, Dacheongchae exhibited higher antimicrobial activities, suggesting synergistic antimicrobial properties from these bioactive compounds in Dacheongchae.

## 1. Introduction

Pakchoi and Tatsoi are subspecies of *Brassica rapa* that are cultivated worldwide due to their economic and nutritional value [1]. These vegetables are regarded as nutritional and functional foods since they contain primary metabolites [1,2], which are considered nutrients for humans, and secondary metabolites [1,3,4], which are considered bioactive compounds for humans.

Glucosinolate is a class of plant secondary metabolites mainly present in *Brassica* vegetables that increases appetite at low levels. These metabolites are subdivided into three subgroups based on their initial precursors. The biosynthesis of aromatic-, aliphatic-, and indolic-group glucosinolates starts with amino phenylalanine, methionine, and tryptophan, respectively [5]. After serial enzyme-related reactions, glucosinolates belonging to each subgroup are biosynthesized and then converted to hydrolysis products (epithionitriles, nitriles, isothiocyanates, or thiocyanates) [6]. These glucosinolates have been reported to reduce cardiometabolic and musculoskeletal disorders, and hydrolyzed products, such as isothiocyanates, have shown preventive effects against psychiatric and neurological disorders and many types of cancer [7].

A carotenoid is a subclass of terpenoid and can be categorized into two subgroups (oxygenated and non-oxygenated groups). The first group, also known as xanthophylls, includes carotenoids containing oxygen atoms (astaxanthin, canthaxanthin, lutein, zeaxanthin, etc.), whereas the second, known as carotenes, purely contains hydrocarbons without oxygen (lycopene, α-carotene, β-carotene, etc.) [8]. Animals are not able to biosynthesize these metabolites; hence, they only acquire them from food sources [9]. Inside the body, these phytochemicals improve human eye health and prevent chronic human diseases [10]. For plants, carotenoids, present in plants, play a main role in light harvest in photosynthetic membranes, flower pollination, and seed dispersal [9].

Phenolics are a class of metabolites with a chemical structure possessing at least one phenol unit, and these metabolites can be subcategorized into subgroups (phenolic acid, flavonoid, tannin, coumarin, lignan, quinone, stilbene, and curcuminoid) [11]. The biosynthesis of these subgroups begins with a precursor (phenylalanine) and then intermediates, and final products belonging to each subgroup can be generated via serial enzymatic reaction. These phenolics are found in higher plants and have significant pharmacological activities (anticancer, antibacterial, and antioxidant) [10].

Targeted metabolite analysis and metabolic profiling are analytical approaches for a variety of plant metabolites. Specifically, the first is to evaluate absolute concentrations of metabolites using specific methods of extraction, isolation, and identification. The other is to detect many metabolites (organic acids, amino acids, carbohydrates, etc.) that are structurally identified, and it is to measure relative metabolite concentrations [12]. For these approaches, high-performance liquid chromatography (HPLC) has been used for target and non-target metabolite analyses. Furthermore, gas-based analytical methods have been widely used because of their powerful isolation, rapid classification, and robust quantification [12,13].

**Figure 1:**
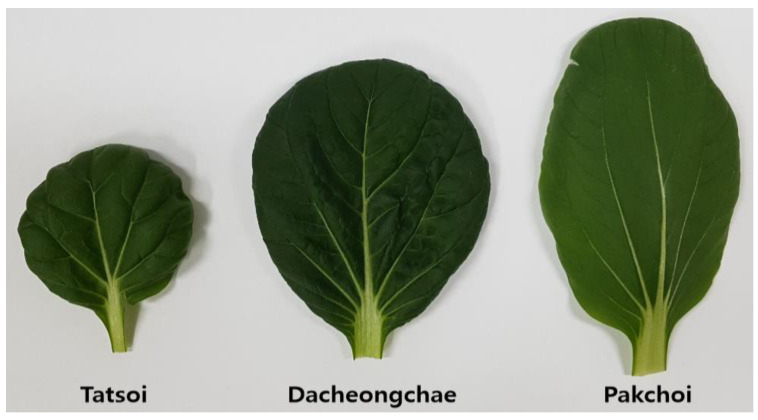
Phenotypes of Tatsoi, Dacheongchae, and Pakchoi used in this study.

The intake of *Brassica* plants has been recommended for human health since these vegetables contain phytochemicals (glucosinolate, carotenoid, and phenolics) that are reported to have health-benefit effects. Therefore, Pakchoi, Tatsoi, and Dacheongchae may be useful food sources. In the present study, these three *Brassica* vegetables were utilized as plant materials (Figure 1). Previously, metabolite analyses of Pakchoi and/or Tatsoi have been described separately. However, there are few studies on Dacheongchae. Furthermore, to date, no studies have previously investigated the difference between primary and secondary metabolites and their antimicrobial effects or documented the correlation between bioactive compounds and their pharmacological activities. Thus, the present study compares metabolites in the three *Brassica* vegetables and describes the connections between identified metabolites and their pharmacological activities.

## 2. Results

### 2.1. Carotenoid Analysis of Tatsoi, Dacheongchae, and Pakchoi

Six carotenoids (zeaxanthin, beta-carotene, alpha-carotene, 9Ζ-beta-carotene, lutein, and 13Z-beta-carotene) were analyzed using HPLC in Pakchoi, Tatsoi, and Dacheongchae (Table 1). Dacheongchae contained the greatest abundance of alpha-carotene, 9Ζ-beta-carotene, beta-carotene, and lutein. In particular, zeaxanthin was identified only in Dacheongchae. Pakchoi had higher levels of lutein, 13Z-beta-carotene, beta-carotene, and 9-cis-beta-carotene than those of Tatsoi. This analysis showed that Dacheongchae is a vegetable with high amounts of carotenoids compared with the other vegetables.

### 2.2. Glucosinolate Analysis of Tatsoi, Dacheongchae, and Pakchoi

Seven glucosinolates (glucoalyssin, progoitrin, gluconapin, neoglucobrassicin, glucobrassicin, glucobrassicanapin, and 4-methoxyglucobrassicin) were analyzed using HPLC in Tatsoi, Dacheongchae, and Pakchoi (Table 2). The highest levels of glucobrassicanapin, 4-methoxyglucobrassicin, and neoglucobassicin were observed in Dacheongchae, and Pakchoi contained the greatest abundance of progoitrin and gluconapin. In addition, the levels of glucoalyssin and glucobrassicin are the lowest in Tatsoi. These findings showed that Dacheongchae and Pakchoi are vegetables with high amounts of glucosinolates compared with Tatsoi.

### 2.3. Phenolic Content Analysis of Tatsoi, Dacheongchae, and Pakchoi

Eight phenolics (catechin, chlorogenic acid, *p*-coumaric acid, caffeic acid, gallic acid, kaempferol, ferulic acid, and *trans*-cinnamic acid) were analyzed using HPLC in Pakchoi, Tatsoi, and Dacheongchae (Table 3). Dacheongchae contained the greatest abundance of chlorogenic acid and caffeic acid, but Tatsoi had the highest levels of *p*-coumaric acid, kaempferol, and ferulic acid. Tatsoi and Dacheongchae contained higher levels of *trans*-cinnamic acid than Pakchoi. Furthermore, although Pakchoi contained relatively lower levels of individual phenolics detected in this study, Dacheongchae contained the highest sum of these eight phenolics, followed by Pakchoi and Tatsoi. These findings are consistent with results from TPC analysis showing that the highest levels were obtained in Dacheongchae, followed by Pakchoi and Tatsoi (Table S1). These analyses highlight that Dacheongchae is a vegetable with high amounts of phenolics compared with the other vegetables.

### 2.4. Metabolic Profiling of Tatsoi, Dacheongchae, and Pakchoi

**Figure 2:**
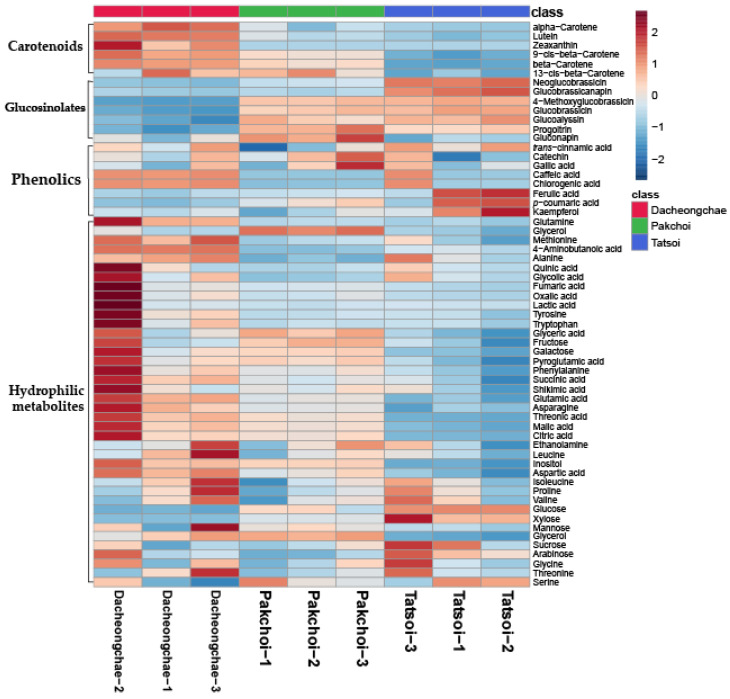
Heatmap representing differences in relative metabolite concentrations of Tatsoi, Dacheongchae, and Pakchoi.

Thirty-nine hydrophilic metabolites, belonging to carbohydrates, amines, amino acids, and organic acids, were identified in Pakchoi, Tatsoi, and Dacheongchae (Table S2). The concentrations of most metabolites identified in the three vegetables were likely to be higher in Dacheongchae (Figure 2). Specifically, the levels of most sugar alcohols and sugars were not significantly different. The levels of galactose and inositol were higher in Pakchoi and Dacheongchae. Tatsoi contained higher levels of xylose and glucose, and Pakchoi had higher levels of glycerol. Among amino acids, the levels of alanine, asparagine, glutamine, 4-aminobutanoic acid, and methionine were higher in Dacheongchae, and the levels of pyroglutamic acid, aspartic acid, and glutamic acid were higher in Dacheongchae and Pakchoi. For tricarboxylic acid (TCA) cycle intermediates, higher levels of fumaric acid, citric acid, and succinic acid were obtained in Dacheongchae, and higher levels of malic acid were found in Dacheongchae and Pakchoi.

**Figure 3:**
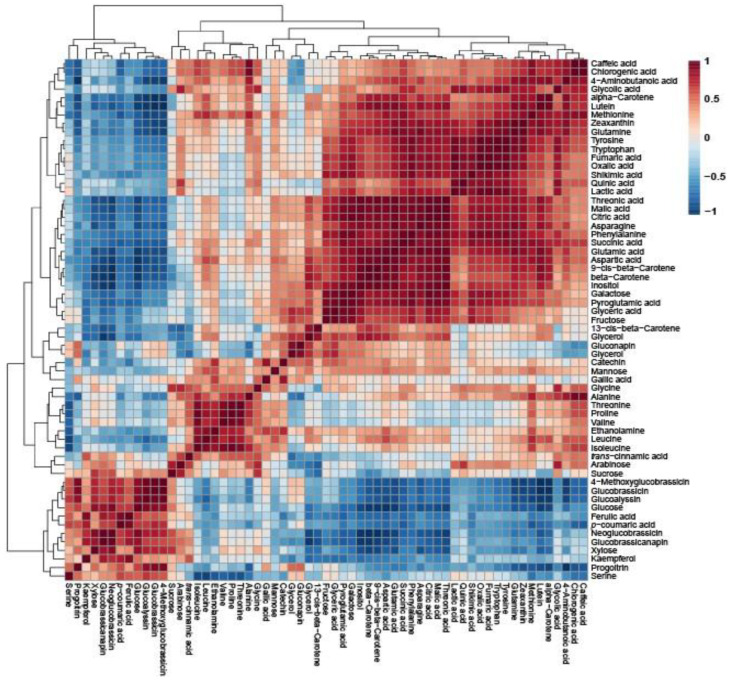
Correlation matrix obtained using 39 metabolites in Tatsoi, Dacheongchae, and Pakchoi. Each square indicates the Pearson’s correlation coefficient of a pair of compounds, and the value of the correlation coefficient is represented by the intensity of the blue or red color, as indicated on the color scale.

Pearson’s correlation analysis of Pakchoi, Tatsoi, and Dacheongcha was performed based on thirty-nine metabolites. Phenylalanine had a positive correlation with caffeic acid, chlorogenic acid, lutein, zeaxanthin, alpha-carotene, beta-carotene, and various amino acids (Figure 3).

**Figure 4:**
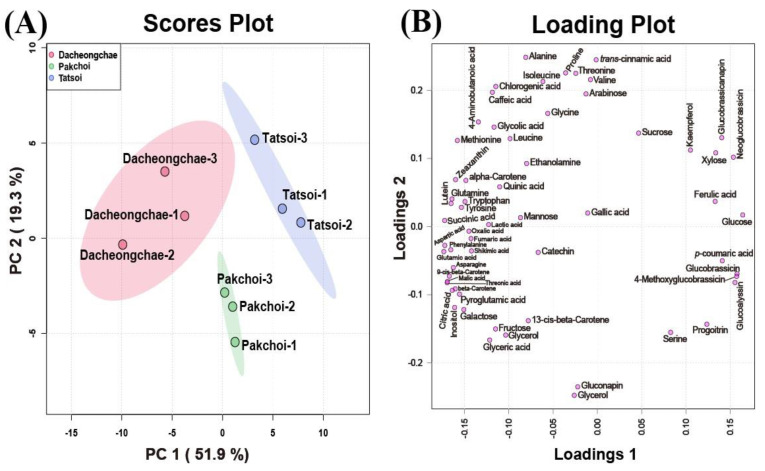

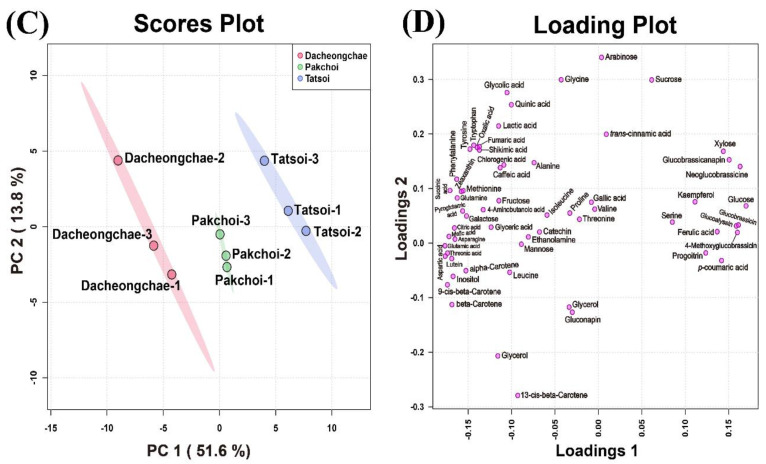
Scores (**A**) and loading plots (**B**) of the principal component analysis (PCA) model obtained from primary and secondary metabolites from Tatsoi, Dacheongchae, and Pakchoi and scores (**C**) and loading plots (**D**) of the partial least squares discriminant analysis (PLS-DA) model obtained from metabolites from Tatsoi, Dacheongchae, and Pakchoi.

These multivariate data were subjected to PCA to investigate the variation in metabolite profiles between Pakchoi, Tatsoi, and Dacheongchae. The PCA results obtained from HPLC analysis of carotenoids, phenolics, and glucosinolates, and GC-TOFMS-based metabolic profiling generated two components, explaining 53.9% for principal component 1 (PC1) and 19.5% for PC2 of the variance, respectively. PC1 revealed that these three vegetable groups were not divided well from each other. Partial least-squares discriminant analysis (PLS-DA) was carried out to maximally isolate groups of Pakchoi, Tatsoi, and Dacheongchae (Figure 4). This revealed clearer isolation among these three vegetables. According to the variables importance in the projection (VIP), 9Ζ-beta-carotene, *p*-coumaric acid, beta-carotene, glucose, neoglucobrassicin, aspartic acid, glutamic acid, lutein, threonic acid, and inositol were the key metabolites affecting this group isolation.

### 2.5. In Vitro Antimicrobial Activities of Tatsoi, Dacheongchae, and Pakchoi

The growth inhibition zone sizes of ten normal pathogens, eight methicillin-resistant pathogens, and one pathogenic yeast using extracts of Tatsoi, Dacheongchae, and Pakchoi at concentrations of 2 mg/disk were estimated using the paper disk method. The extracts of these cultivars showed antimicrobial abilities against *B. cereus* (KCTC 3624), *E. coli* (KCTC 1682), *P. mirabilis* (KCTC 2510), *P. aeruginosa* (KCCM 11803), *C. albicans* (ATCC 28367), and *P. aeruginosa* (0225, 0254, 0826, P01827, 1731, 1378, 1113, and P01828), while the growth inhibition of *S. paratyphi* (KCCM 41577) was observed only in the extracts of Dacheongchae (Table 4). In addition, the antimicrobial effects of *V. parahaemolyticus* (KCTC 2471), *S. mutans* (KCTC 3065), *S. aureus* (KCTC 3881), and *M. luteus* (KCTC 3063) were not found in the extracts of these three cultivars. Specifically, Dacheongchae extracts possessed the highest inhibition effects against *P. aeruginosa* (KCCM 11803) and methicillin-resistant *P. aeruginosa* (0254, 1113, 1378, 1731, and P01828), Tatsoi extracts had the highest effect against *P. mirabilis* (KCTC 2510), and Pakchoi extracts showed the highest effect against *E. coli* (KCTC 1682). Furthermore, both extracts of Dacheongchae and Tatsoi showed higher abilities against *B. cereus* (KCTC 3624), *C. albicans* (ATCC 28367), and *P. aeruginosa* (0225, 1113, and P01827), and extracts of Pakchoi and Tatsoi showed higher abilities against *B. cereus* (KCTC 3624), *C. albicans* (ATCC 28367), and *P. aeruginosa* (0225, 1113, and P01827) (Figure S4).

## 3. Discussion

According to the metabolite profiles of Tatsoi, Dacheongchae, and Pakchoi, the endogenous levels of most amino acids were likely to be high in Dacheongchae. Particularly, this cultivar contained significantly higher levels of amino acids such as alanine, asparagine, glutamine, pyroglutamic acid, 4-aminobutanoic acid, methionine, aspartic acid, and glutamic acid and TCA cycle intermediates including malic acid, succinic acid, fumaric acid, and citric acid. Furthermore, the strong positive correlation between amino acids and TCA cycle intermediates was confirmed in these cultivars. This may be because the TCA cycle provides essential precursors for amino acid biosynthesis (oxaloacetate and α-ketoglutarate). These findings were consistent with previous studies reporting a strong positive correlation between the majority of amino acids and TCA cycle intermediates in oval- and rectangular-shaped Chinese cabbage cultivars [14], a positive correlation between most amino acids and TCA cycle intermediates, including succinic acid, citric acid, and malic acid, detected in 10-day-old *Arabidopsis* seedlings [15], and a positive correlation between the amino acid pool and TCA cycle intermediates in tobacco plants [16].

According to secondary metabolite analysis of Pakchoi, Tatsoi, and Dacheongchae, eight phenolics (chlorogenic acid, gallic acid, *p*-coumaric acid, ferulic acid, caffeic acid, catechin, *trans*-cinnamic acid, and kaempferol), six carotenoids (13*Z*-beta-carotene, lutein, zeaxanthin, alpha-carotene, 9*Ζ*-beta-carotene, and beta-carotene), and seven glucosinolates (progoitrin, gluconapin, glucoalyssin, glucobrassicin, glucobrassicanapin, neoglucobrassicin, and 4-methoxyglucobrassicin) were detected in Pakchoi, Tatsoi, and Dacheongchae in this study. These findings are consistent with previous studies reporting the identification of glucosinolates, carotenoids, and phenolics in these cultivars. For instance, Liang et al., 2018, reported the identification of glucosinolates (gluconapin, glucobrassicanapin, progoitrin, glucobrassicin, glucoalyssin, 4-methoxyglucobrassicin, and neoglucobrassicin) in Pakchoi [17]. Furthermore, phenolic compounds (catechin, caffeic acid, chlorogenic acid, *p*-coumaric acid, *trans*-cinnamic acid, ferulic acid, and kaempferol) in green, pale green, white, and purple Pakchoi [18,19] and carotenoids (zeaxanthin, 13Z-9Ζ-beta-carotene, beta-carotene, alpha-carotene, lutein, and beta-carotene) in green, pale green, and white Pakchoi [19] were identified. Furthermore, Pannico et al., 2020, reported that kaempferol derivatives, chlorogenic acid, caffeic acid, coumaric acid, and ferulic acid are founds in Tatsoi microgreens [4,20]; beta-carotene, alpha-carotene, violaxanthin, and neoxanthin are found in Tatsoi microgreens [3]; and alpha-carotene, neoxanthin, beta-carotene, violaxanthin, lutein, and zeaxanthin are found in Tatsoi baby leaves [21].

Secondary metabolites can be generated from primary metabolites. Phenolic compounds can be biosynthesized from phenylalanine, and the production of indolic glucosinolates and aliphatic glucosinolates can begin with tryptophan and methionine, respectively. In the current study, a positive correlation was observed between secondary metabolites (phenolics, indolic glucosinolates, and aliphatic glucosinolates, respectively) and their precursors (phenylalanine, tryptophan, and methionine, respectively) in Pakchoi, and Tatsoi, and Dacheongchae. These results agree with those of prior studies revealing a correlation between secondary metabolites and their precursors. There was a positive correlation between phenylalanine and phenolic compounds in the roots and leaves of Chinese cabbage (*B. rapa* subsp. *pekinensis*). Positive correlations were found between indolic glucosinolates and tryptophan in the roots and between aliphatic glucosinolates and methionine in the leaves [9]. Similarly, phenylalanine was positively correlated with phenolics in the oval- and rectangular-shaped Chinese cabbage cultivars [9], and methionine and tryptophan were positively correlated with aliphatic and indolic glucosinolates in the shoots of S-deficient Chinese cabbage [22].

To determine the correlation between metabolites quantified in Pakchoi, Tatsoi, and Dacheongchae, HCA was performed using Pearson’s correlation results on the multivariate data. Specifically, glutamate and aspartate were recognized as common precursors for the amino acids of the glutamate family (e.g., glutamate, glutamine, arginine, proline, and 4-aminobutyric acid) [23,24] and aspartate family (e.g., aspartate, asparagine, methionine, and threonine) [23] in the plant amino acid biosynthesis. The shikimic acid pathway provides carbon skeletons for the amino acids of the aromatics (tryptophan, phenylalanine, and tyrosine) [25]. Thus, this study indicates that glutamate and aspartate were positively correlated with the other amino acids. Specifically, glutamic acid was strongly positively correlated with glutamine (*r* = 0.92437, *p* = 0.00036), pyroglutamate (*r* = 0.86568, *p* = 0.00256), and 4-aminobutyric acid (*r* = 0.72313, *p* = 0.0277), and aspartic acid was highly positively correlated with asparagine (*r* = 0.8671, *p* = 0.00247) and methionine (*r* = 0.84014, *p* = 0.00458). Furthermore, shikimic acid had positive correlations with aromatic amino acids, such as tryptophan (*r* = 0.83958, *p* = 0.004637), phenylalanine (*r* = 0.93557, *p* = 0.00021), and tyrosine (*r* = 0.88876, *p* = 0.001354). Amino acid metabolism is closely linked with the TCA cycle. TCA intermediates (oxaloacetate and α-ketoglutarate) can be used to form aspartate and glutamate, respectively. In the TCA cycle, oxaloacetate can be synthesized to citrate by citrate synthase, and citrate is isomerized to isocitrate by aconitase. Afterwards, isocitrate dehydrogenase catalyzes the dehydrogenation of isocitrate to oxalosuccinate and the decarboxylation of oxalosuccinate to α-ketoglutarate. Furthermore, α-ketoglutarate undergoes oxidative decarboxylation by a dehydrogenase complex to form succinyl-CoA, and then succinyl-CoA can be converted into succinate by succinate thiokinase. Then, succinate dehydrogenase catalyzes the dehydrogenation of succinate to fumarate, and fumarase catalyzes the oxidation of fumarate to malate. Finally, malate becomes oxaloacetate by malate dehydrogenase [26]. Citrate was positively correlated with malic acid (*r* = 0.99291, *p* = 0.0000000979), succinic acid (*r* = 0.90376, *p* = 0.00083), and fumaric acid (*r* = 0.88138, *p* = 0.00168) in this study. Citric acid is recognized as a precursor for α-ketoglutarate, which begins the biosynthesis of the glutamate family amino acid, and fumarate is used as a precursor for oxaloacetate, which begins the biosynthesis of the aspartate family amino acid. Thus, this study indicated that citrate was strongly positively correlated with glutamine (*r* = 0.94562, *p* = 0.00012), glutamine (*r* = 0.85682, *p* = 0.00304), and pyroglutamate (*r* = 0.94324, *p* = 0.00014), and fumarate had a strong positive correlation with asparagine (*r* = 0.95581, *p* = 0.00287). Methionine is a precursor for aliphatic glucosinolates, such as progoitrin, glucoalyssin, glucobrassicanapin, etc., and tyrosine is a precursor for indolic glucosinolates, including glucobrassicin, 4-methoxyglucobrassicin, neoglucobrassicin, etc. In this study, methionine was positively correlated with progoitrin, glucoalyssin, and glucobrassicanapin, and tyrosine was positively correlated with glucobrassicin, neoglucobrassicin, and 4-methoxyglucobrassicin, as shown in Figure 3. In addition, phenylalanine had positive correlations with chlorogenic acid, caffeic acid, and catechin.

The major difficulty of this study is the accurate metabolite identification. One way to overcome this limitation has been the use of different tools to improve accuracy and reproducibility in metabolomics research. Though, in this study, GC-TOFMS has been used for the metabolite analysis, nuclear magnetic resonance (NMR) can be a good tool to identify various compounds (carbohydrate, amino acid, organic acid, fatty acid, amine, ester, ether, and lipid) from plant samples [27]. For example, NMR spectroscopy has been performed to estimate the metabolite differences between various cultivars and developmental stages in *B. rapa* subsp. *oleifera* [28], *B. rapa* var. *rapa* [29], *B. rapa* ssp. *Pekinensis* [30]. However, the metabolomic analysis of *B. rapa* ssp. *narinosa*, *B. rapa* var. *narinosa* × *chinensis*, and *B. rapa* ssp. *chinensis* has been not carried out by NMR spectroscopy yet. Therefore, further study is needed to perform NMR metabolomics in these three cultivars.

The present study revealed that the extracts of Pakchoi, Tatsoi, and Dacheongchae possessed strong antimicrobial effects against *E. coli* (KCTC 1682), *B. cereus* (KCTC 3624), *P. mirabilis* (KCTC 2510), *P. aeruginosa* (KCCM 11803), *C. albicans* (ATCC 28367), and *P. aeruginosa* (0225, 0254, 0826, P01827, 1113, 1731, 1378, and P01828, respectively). The findings of previous studies describe the antimicrobial properties of *B. rapa* varieties. For example, Chinese cabbage extraction inhibited *C. albicans*, *S. aureus*, and *E. coli* [31], and purple-colored and typical Chinese cabbage cultivars showed antimicrobial effects against *B. cereus*, *P. aeruginosa*, *S. aureus*, and *E. coli* and methicillin-resistant *P. aeruginosa* (0826, 0225, 0254, 1113, 1731, 1827, and 1828) [32]. The inhibition activity against *E. coli*, *S. aureus*, *P. aeruginosa*, and *C. albicans* was confirmed in the extracts of Rapa Catozza Napoletana (*B. rapa* L. var. *rapa*) [33]. Furthermore, *Brassica* crops showed strong antimicrobial effects against various pathogens. Varghese (2015) reported that extracts of red cabbage (*B. oleracea* var. *capitata*) had inhibitory activity against *Enterococcus faecalis*, *E. coli*, *S. aureus*, *M. luteus*, *P. aeruginosa*, and vancomycin-resistant *E. faecalis* [34]. Additionally, broccoli extracts (*B. oleracea* var. *italica*) were reported to have antibacterial effects against *B. cereus* [35], and extracts of savoy cabbage (*B. oleracea* var. *sabauda*) and white cabbage (*B. oleracea* var. *capitata*) showed inhibitory activity against *P. aeruginosa* [36].

Moreover, higher antimicrobial and antioxidant properties were confirmed in Dacheongchae extracts compared with the extracts of Pakchoi and Tatsoi. This might be due to higher levels of secondary metabolites (total phenolics, phenolic compounds, glucosinolates, and carotenoids) in Dacheongchae. Specifically, phenolic compounds, such as polyphenol, have been reported to provide antioxidant effects through their antioxidant capacity, such as reactive oxygen species (ROS) removal and ROS formation inhibition [37]. Carotenoids can be grouped into carotenes, including lycopene, β-carotene, and α-carotene, and xanthophylls, including lutein, β-cryptoxanthin, and zeaxanthin. These carotenoids contain a conjugated double bond structure providing resistance to oxidative stress [38], and the breakdown compounds of glucosinolates have both antioxidant and pro-oxidant capacities, modulating the oxidant/antioxidant balance at the cellular level [39]. In addition to antioxidant properties, phenolics have been reported to have strong antimicrobial effects through their potential mechanisms, including hindrance of enzymatic activity, DNA structure alteration, disruption of ribosomal translation, and induction of membrane disruption, depolarization, or cellular content leakage [40]. Glucosinolate derivatives, isothiocyanates (ITCs), possess antimicrobial effects via disruption of bacteria membranes, metabolic alteration, and stress response activation [41]. Antimicrobial mechanisms of carotenoids are little known. Astaxanthin and fucoxanthin, belonging to the carotenoid class, have potential antibacterial mechanisms, such as oxidative phosphorylation alteration, efflux pump modulation, and inhibition of biofilm formation, oxygen uptake, virulence factors, or nucleic acid [42].

Considering various studies on antimicrobial properties in extracts of *Brassica* family vegetables, antimicrobial activities against pathogens varied depending on the species and variety of *Brassica* and different species of the pathogens. Therefore, hybridization can be a good way for *Brassica* crop improvement.

## 4. Materials and Methods

### 4.1. Plant Materials

Tatsoi, Dacheongchae, and Pakchoi have been purchased at the ASIA SEED KOREA Co., Ltd. (Seoul, Republic of Korea), and they were cultivated under the growth chamber for three weeks. The bed soil used in this study was purchased from the NONGWOOBIO Co., Ltd. (Suwon-si, Republic of Korea) and was composed of cocofeat (49.876%), peat moss (25%), perlite (12%), vermiculite (7%), zeolite (6%), wood vinegar (0.004%), fertilizer (0.11%), and humectant (0.01%). Three independent plants were prepared for this study. The harvested plants were immediately frozen with N2, lyophilized, and ground for further studies.

### 4.2. Sample Preparation for HPLC Chemical Analysis

In this study, 2 mL of 70% methanol (*v*/*v*) was added to 100 mg of freeze-dried powder of the three *B. rapa* crops, followed by vortexing for 10 s and sonication for 60 min. After centrifugation at 11,000× *g* for 15 min, the obtained supernatant was subject to syringe filtration for HPLC analysis of phenolics [9,14]. For quantification of carotenoids, 1 mL of 0.1% ascorbic acid-ethanol (*w*/*v*) was added to 0.1 g of freeze-dried powder, followed by incubation for 20 min at 85 °C. A volume of 0.12 mL of 80% potassium hydroxide (*w*/*v*) was added, and then the samples were incubated at 85 °C for 15 min. After standing on ice for 10 min, 50 μL of beta-apo-8′-carotenal (25 mg/L), as an internal standard, was added, followed by the addition of 1.5 mL of cold water and 1.5 mL of hexane. The mixture was thoroughly mixed and then centrifuged at 11,000× *g* for 25 min at 4 °C. The aqueous solution was collected in a sterilized tube. These methods of extraction were performed in triplicate. Subsequently, nitrogen (N2) was used to dry the collected aqueous solutions, and 0.3 mL of a dichloromethane–methanol mixture (50% *v*/*v*) was used to re-dissolve the dried samples, followed by syringe filtration into a sterilized vial [9,14]. Prior to quantification of glucosinolates, 1500 μL of boiling 70% methyl alcohol (*v*/*v*) at 70 °C was added to 100 mg of freeze-dried powder of the three *B. rapa* crops, and samples were left to stand at 70 °C for 10 min in a bath. After centrifugation for 20 min at 11,000× *g*, the supernatants were collected in sterilized tubes. The residues were re-extracted twice using the method described above. The collected extract of these three cultivars was loaded onto a column packed with DEAE-Sephadex A-25 (Sigma-Aldrich, St. Louis, MO, USA), and then 75 µL of arylsulfatase solution were added for desulfation, followed by elution of desulfoglucosinolates by adding 1.5 mL of water. The final extracts were syringe-filtrated into sterilized vials [9,19].

### 4.3. HPLC Analysis of Phenolics, Carotenoids, and Glucosinolates

Phenolics were quantitated in Tatsoi, Dacheongchae, and Pakchoi, respectively, with a previously reported method [9,18]. The HPLC analysis equipment and operation condition were the same as those reported in a previous study (Table S3) [18,42]. Peak was identified in comparison with the retention time of each standard chemical and spike test, and its quantification was carried out using calibration curves for gallic acid, *p*-coumaric acid, catechin, ferulic acid, chlorogenic acid, caffeic acid, kaempferol, and *trans*-cinnamic acid. Carotenoids were quantitated in Tatsoi, Dacheongchae, and Pakchoi using a previous method [9]. The HPLC analysis equipment and operation conditions were the same as those reported in a previous study (Table S4) [9,19]. Quantification of carotenoids was carried out using calibration curves of 9-*cis*-β-carotene, lutein, 9-*cis*-beta-carotene, alpha-carotene, zeaxanthin, beta-carotene, and 13-*cis*-beta-carotene. Desulfoglucosinolates were quantitated in Tatsoi, Dacheongchae, and Pakchoi using a previous method [9,19]. The HPLC analysis equipment, operation conditions, and quantification of desulfoglucosinolates were the same as those reported in a previous study (Table S5) [9,19].

### 4.4. Gas Chromatography-Time-of-Flight Mass Spectrometry (GC-TOFMS) Analysis

Hydrophilic metabolites were identified and quantitated in Tatsoi, Dacheongchae, and Pakchoi using a previously reported method [9]. One milliliter of a water/chloroform/methanol = 1:1:2.5 (*v*/*v*/*v*) and sixty microliters of ribitol (0.2 g/L), as an internal standard, were added to ten milligrams of freeze-dried powder of the three *B. rapa* crops (Dacheongchae, Pakchoi, and Tatsoi). Samples were then shaken at 1200× *g* for 30 min at 37 °C. After centrifugation at 11,000× *g* for 10 min, 800 μL of the polar phase was added to 0.4 mL of water and then evaporated. Subsequently, the addition of 0.04 mL of hydrochloride/pyridine was added for derivatization. These mixtures were then mixed at 37 °C for 2 h and spun down, and 0.08 mL of N-methyl-N-(trimethylsilyl)trifluoroacetamide was added. This mixture was incubated at 37 °C for 30 min and then transferred to a sterilized vial for GC-TOFMS analysis. The GC-TOFMS analysis equipment and operation conditions were the same as those reported in a previous study [9]. Chroma-TOF software (version 4.72, LECO, St. Joseph, MI, USA) and selected ions were used for the quantification and identification of hydrophilic metabolites [9].

### 4.5. In Vitro Antimicrobial Properties Assay

Antimicrobial screening of the Tatsoi, Dacheongchae, and Pakchoi extracts was carried out using a previously reported method [43]. Then, 20 mL of methanol was added to 1 g of freeze-dried powder from Tatsoi, Dacheongchae, and Pakchoi, followed by shaking for 1 day and centrifugation at 11,000× *g* for 20 min. After filtration of the supernatant via filter paper, the crude extract was evaporated completely and then re-dissolved in methanol to a concentration of 100 mg/mL for a further antimicrobial assay. Pathogens were cultured at 100 rpm and 30 °C to an OD600 of 1.0. *Bacillus cereus*, *Escherichia coli*, *Pseudomonas aeruginosa*, *Staphylococcus aureus*, *Salmonella paratyphi* C, *Proteus mirabilis* (KCTC 3624, KCCM 11803, KCTC 3881, KCTC 1682, KCCM 41577, and KCTC 2510), and *P. aeruginosa* (0225, 0254, 1113, P01827, 1378, 0826, 1731, and P01828) were cultured in nutrient broth (NB). *Micrococcus luteus*, *Streptococcus mutans*, and *Vibrio parahaemolyticus* (KCTC 3063, KCTC 3065, and KCTC 2471) were cultured in No. 2 enriched NB, marine agar, and brain heart infusion broth. *Candida albicans* (ATCC 28367) was cultured in yeast malt broth. Next, 100 mL of each microbial culture (OD600 of 1.0) was mixed with the warm agar medium (~40 °C), and then the mixture was loaded on a sterilized Petri dish. After solidification, three sterilized fresh paper disks that were saturated with extract containing either Dacheongchae, Pakchoi, or Tatsoi (2.0 mg/disk) were placed on the solid agar medium. Each Petri dish was then incubated at the proper temperature for the microbes for 1 day. Diameters of the resulting zones of growth inhibition were measured.

### 4.6. Statistical Analysis

SAS software version 9.4 (SAS Institute Inc., Cary, NC, USA) was used for Duncan’s multiple range test (DMRT). MetaboAnalyst 6.0 “http://www.metaboanalyst.ca/ (accessed on 10 January 2025)” was carried out for analysis of the metabolite data, including Pearson correlation analysis, principal component analysis, and heat maps.

## 5. Conclusions

Conclusively, metabolomic analyses and biological activity bioassays were carried out using Tatsoi, Dacheongchae, and Pakchoi, respectively. Dacheongchae contained a greater abundance of amino acids as well as a greater abundance of phenolic compounds and carotenoids compared with Pakchoi and Tatsoi. Consistent with the highest levels of phytochemicals, the highest inhibitory activities against *B. cereus* (KCTC 3624), *P. mirabilis* (KCTC 2510), *E. coli* (KCTC 1682), *P. aeruginosa* (KCCM 11803), *C. albicans* (ATCC 28367), and *P. aeruginosa* (0225, 0254, 0826, 1113, 1378, 1731, P01827, and P01828, respectively) were confirmed in Dacheongchae. Regarding the importance of nutrients and functionality from plant food products, Dacheongchae could serve as a significant contribution to the food industry due to its great abundance of primary metabolites (free amino acids) and secondary metabolites (carotenoids and phenolics). Thus, the present study provides information on nutrients, bioactive compounds, and antioxidant and antimicrobial activities in these three cultivars.

## Figures and Tables

**Table 1:** Carotenoid analysis in Tatsoi, Dacheongchae, and Pakchoi (μg g^−1^ dry weight).

Compounds (μg g^−1^ dw)	Tatsoi	Dacheongchae	Pakchoi
Lutein	259.02 ± 25.06 c	668.71 ± 18.28 a	352.77 ± 38.37 b^1^
Zeaxantin	N.D.	4.29 ± 1.54 a	data N.D.^2^
13Z-β-Carotene	32.78 ± 4.65 b	61.53 ± 17.32 a	64.29 ± 9.37 a
α-Carotene	7.13 ± 0.28 b	13.98 ± 0.92 a	7.95 ± 1.44 b
β-Carotene	298.14 ± 8.75 c	683.42 ± 19.08 a	554.71 ± 20.00 b
9Ζ-β-Carotene	34.77 ± 3.94 c	104.61 ± 8.33 a	76.76 ± 2.18 b
Total	631.85 ± 30.80 c	1536.54 ± 31.18 a	1056.48 ± 58.78 b

^1^ Means with different letters (a–c) are significantly different at *p* < 0.05 using DMRT. ^2^ N.D., not detected.

**Table 2:** Glucosinolates analysis in Tatsoi, Dacheongchae, and Pakchoi (μmol g^−1^ dry weight).

Compounds (μmol g^−1^ dw)	Tatsoi	Dacheongchae	Pakchoi
Progoitrin	0.47 ± 0.04 c	0.77 ± 0.02 b	0.88 ± 0.07 a^1^
Glucoalyssin	0.34 ± 0.03 b	0.58 ± 0.03 a	0.54 ± 0.04 a
Gluconapin	3.87 ± 0.36 b	3.59 ± 0.22 b	4.82 ± 0.29 a
Glucobrassicanapin	1.18 ± 0.06 b	2.32 ± 0.12 a	1.26 ± 0.05 b
Glucobrassicin	0.08 ± 0.02 b	0.42 ± 0.03 a	0.37 ± 0.03 a
4-Methoxyglucobrassicin	0.05 ± 0.01 c	0.84 ± 0.03 a	0.77 ± 0.03 b
Neoglucobrassicin	0.05 ± 0.01 b	0.39 ± 0.01 a	0.14 ± 0.01 b
Total	6.04 ± 0.43 b	8.91 ± 0.40 a	8.79 ± 0.44 a

^1^ Means with different letters (a–c) are significantly different at *p* < 0.05 using DMRT.

**Table 3:** Phenolics analysis in Tatsoi, Dacheongchae, and Pakchoi (μg g^−1^ dry weight).

Compounds (μg g^−1^ dw)	Tatsoi	Dacheongchae	Pakchoi
Gallic acid	16.93 ± 0.21 a	16.91 ± 0.22 a	17.07 ± 0.39 a^1^
Catechin	661.76 ± 41.62 a	709.72 ± 32.31 a	738.99 ± 41.67 a
Chlorogenic acid	116.49 ± 0.78 b	199.51 ± 2.09 a	107.56 ± 1.13 c
Caffeic acid	36.02 ± 0.41 b	105.05 ± 1.82 a	33.92 ± 0.19 b
*p*-coumaric acid	7.11 ± 0.18 a	3.14 ± 0.19 c	4.78 ± 0.52 b
Ferulic acid	7.24 ± 0.53 a	1.89 ± 0.32 b	2.50 ± 0.15 b
*trans*-cinnamic acid	1.46 ± 0.13 a	1.37 ± 0.09 ab	1.18 ± 0.15 b
Kaempferol	79.38 ± 0.55 a	77.90 ± 0.10 b	77.72 ± 0.36 b
Total	882.33 ± 43.89 c	1115.49 ± 37.14 a	983.72 ± 44.56 b

^1^ Means with different letters (a–c) are significantly different at *p* < 0.05 using DMRT.

**Table 4:** Antibacterial activity of methanol extracts of the three different Chinese cabbage extracts, respectively.

Group	Bacterial Strains	Zone of Inhibition (mm)		
		Tatsoi	Dacheongchae	Pakchoi
Pathogens	*Vibrio parahaemolyticus* (KCTC 2471)	—	—	— ^1^
	*Streptococcus mutans* (KCTC 3065)	—	—	—
	*Staphylococcus aureus* (KCTC 3881)	—	—	—
	*Micrococcus luteus* (KCTC 3063)	—	—	—
	*Bacillus cereus* (KCTC 3624)	11.5–12	11.5–12	10.5–11
	*Escherichia coli* (KCTC 1682)	9.5–10	12–12.5	12.5–13
	*Proteus mirabilis* (KCTC 2510)	13–14	11–12	10–10.5
	*Salmonella paratyphi C* (KCCM 41577)	—	11–12	—
	*Pseudomonas aeruginosa* (KCCM 11803)	11.5–12	15–16	12–13
Pathogenic yeast	*Candida albicans* (ATCC 28367)	11.5–12	11.5–12.5	11–11.5
Multidrug-resistant pathogens	*Pseudomonas aeruginosa* (0225)	11–11.5	11–11.5	9.5–10
	*Pseudomonas aeruginosa* (0254)	12–12.5	14–15	11.5–12
	*Pseudomonas aeruginosa* (0826)	10–10.5	9–10	10.5–11
	*Pseudomonas aeruginosa* (1113)	12–13	12–13	10–11
	*Pseudomonas aeruginosa* (1378)	11–12	12–13	11–11.5
	*Pseudomonas aeruginosa* (1731)	10.5–11	12–13	10–10.5
	*Pseudomonas aeruginosa* (P01827)	11–12	11–12	9–10
	*Pseudomonas aeruginosa* (P01828)	9–9.5	13–14	9.5–10

^1^—not detected.

## Data Availability

Data are contained within this article and Supplementary Materials.

## Supplementary Materials

The following supporting information can be downloaded at: https://www.mdpi.com/article/10.3390/molecules30081693/s1. Table S1: Phenolic content analysis in Tatsoi, Dacheongchae, and Pakchoi (μg/g); Table S2: Hydrophilic metabolites in Tatsoi, Dacheongchae, and Pakchoi; Table S3: The equipment and operation condition of HPLC analysis for phenolics; Table S4: The equipment and operation condition of HPLC analysis for carotenoids; Table S5: The equipment and operation condition of HPLC analysis for glucosinolates; Figure S1: HPLC chromatograms of carotenoids extracted from Pakchoi (**a**), Tatsoi (**b**), and Dacheongchae (**c**); Figure S2: HPLC chromatograms of glucosinolates extracted from Tatsoi (**a**), Dacheongchae (**b**), and Pakchoi (**c**); Figure S3: GC-TOF-MS chromatograms of hydrophilic metabolites obtained from Pakchoi (**a**), Tatsoi (**b**), and Dacheongchae (**c**); Figure S4: Representative images showing antibacterial activities of methanol extracts of Tatsoi, Dacheongchae, and Pakchoi (right, extracts of Tatsoi; middle, extracts of Dacheongchae; and left, extracts of Pakchoi).
