# Supplementary material for: Metabolite Profiling and Antimicrobial Activities of Brassica rapa ssp. narinosa (Tatsoi), Brassica rapa var. narinosa × chinensis (Dacheongchae), and Brassica rapa ssp. chinensis (Pakchoi)
Source: Molecules. 2025 Apr 10;30(8):1693. doi: 10.3390/molecules30081693 (PMC12029358; doi:10.3390/molecules30081693)
Supplement: Supplementary file 1 [file molecules-30-01693-s001.zip › molecules-3401797-supplementary.pdf]

Table S1. Phenolic content analysis in Tatsoi, Dacheongchae, and Pakchoi (μg/g)

	**Tatsoi**	**Dacheongchae**	**Pakchoi**
**TPC**	48.91 ± 0.85 b	65.51 ± 3.84 a	46.79 ± 1.55 b[1]

[1]Means with different letters (a and b) are significantly different at $p < 0.05$ using DMRT

Table S2. Hydrophilic metabolites in Tatsoi, Dacheongchae, and Pakchoi

Intensity (ratio/g)	Tatsoi	Dacheongchae	Pakchoi
Arabinose	4.41 ± 0.60 a	3.90 ± 0.95 ab	2.94 ± 0.29 b[1]
Fructose	838.94 ± 101.29 a	1022.87 ± 200.71 a	1088.39 ± 30.16 a
Glycerol	101.07 ± 32.70 c	227.42 ± 27.19 b	438.33 ± 17.01 a
Xylose	2.08 ± 0.74 a	0.00 ± 0.00 c	0.89 ± 0.04 b
Sucrose	104.58 ± 27.11 a	75.22 ± 18.15 a	77.34 ± 10.82 a
Inositol	80.67 ± 4.89 b	144.89 ± 15.74 a	130.02 ± 0.93 a
Galactose	6.64 ± 0.48 b	10.54 ± 2.72 a	9.82 ± 0.12 ab
Glucose	235.60 ± 1.73 a	134.73 ± 3.86 c	188.16 ± 20.95 b
Mannose	0.70 ± 0.06 a	1.01 ± 0.54 a	0.90 ± 0.05 a
Alanine	28.83 ± 17.94 a	42.39 ± 5.28 a	6.42 ± 2.94 b
Asparagine	0.49 ± 0.15 b	2.01 ± 0.69 a	1.07 ± 0.05 b
Serine	26.40 ± 7.81 a	17.11 ± 8.93 a	25.98 ± 5.74 a
Proline	5.99 ± 3.69 a	7.21 ± 4.79 a	3.30 ± 2.00 a
Valine	21.66 ± 9.20 a	21.33 ± 9.41 a	15.82 ± 6.34 a
Glutamine	1.59 ± 0.63 b	10.88 ± 3.81 a	2.48 ± 0.23 b
Pyroglutamic acid	87.10 ± 32.71 b	182.78 ± 56.55 a	164.83 ± 7.06 a
Isoleucine	13.66 ± 5.08 a	17.41 ± 6.26 a	10.66 ± 4.57 a
Leucine	6.57 ± 3.03 a	13.85 ± 6.51 a	7.84 ± 3.76 a
4-Aminobutanoic acid	20.11 ± 3.24 b	48.76 ± 0.62 a	10.10 ± 1.44 c
Glycine	8.76 ± 1.54 a	8.75 ± 1.25 a	7.99 ± 0.83 a
Methionine	2.2 ± 0.73 b	3.82 ± 0.44 a	2.25 ± 0.18 b
Tryptophan	1.01 ± 0.39 a	3.59 ± 2.36 a	1.12 ± 0.17 a
Phenylalanine	4.64 ± 0.79 b	7.59 ± 1.95 a	6.06 ± 0.26 ab
Tyrosine	5.05 ± 1.15 a	10.91 ± 4.94 a	5.55 ± 0.34 a
Threonine	11.18 ± 2.80 a	11.77 ± 3.62 a	9.22 ± 1.10 a
Aspartic acid	19.77 ± 8.59 c	65.60 ± 7.28 a	44.97 ± 5.72 b
Glutamic acid	16.30 ± 4.14 c	48.63 ± 8.17 a	31.06 ± 1.48 b
Glyceric acid	12.88 ± 1.18 b	16.49 ± 2.96 ab	17.58 ± 0.59 a
Glycolic acid	8.81 ± 1.84 a	11.05 ± 3.23 a	6.82 ± 0.38 a
Lactic acid	7.57 ± 1.40 a	31.11 ± 37.08 a	6.70 ± 1.27 a
Malic acid	151.97 ± 63.58 b	1176.92 ± 417 a	767.85 ± 28.13 a
Oxalic acid	1.00 ± 0.19 a	4.53 ± 3.70 a	1.70 ± 0.40 a
Succinic acid	17.8 ± 4.39 b	29.46 ± 4.89 a	22.09 ± 0.80 ab
Threonic acid	25.6 ± 1.81 c	98.63 ± 23.07 a	69.53 ± 2.74 b
Shikimic acid	0.71 ± 0.22 a	1.11 ± 0.42 a	0.91 ± 0.08 a
Fumaric acid	1.87 ± 0.78 a	9.84 ± 8.87 a	3.76 ± 0.44 a

Citric acid	5.69 ± 2.22 b	99.87 ± 47.93 a	64.82 ± 5.4 a
Quinic acid	0.95 ± 0.21 a	1.31 ± 0.68 a	0.77 ± 0.06 a
Ethanolamine	16.67 ± 5.74 a	21.81 ± 6.16 a	19.77 ± 5.50 a

[1]Means with different letters (a–c) are significantly different at $p < 0.05$ using DMRT

Table S3. The equipment and operation condition of HPLC analysis for phenolics

Equipment	NS-4000 (Futecs, Daejeon, Korea)
Column	Optimapak C18 column (250 mm × 4.6 mm, 5 μm; RStech, Daejon, Korea)
Oven Temperature	30 °C
Detector	UV-Vis
Detector Wavelength	275 nm
Flow Rate	1.0 mL/min
Mobile Phase	0.2% (*v/v*) acetic acid, solvent A and methanol, solvent B
Gradients	solvent A 95%; 1 min,<br>solvent A 85%; 4 min,<br>solvent A 85%; 9 min,<br>solvent A 80%; 14 min,<br>solvent A 80%; 24 min,<br>solvent A 70%; 54 min,<br>solvent A 55%; 55 min,<br>solvent A 55%; 65 min,<br>solvent A 44%; 75 min,<br>solvent A 40%; 77 min,<br>solvent A 40%; 79 min,<br>solvent A 20%; 80 min,<br>solvent A 20%; 90 min,<br>solvent A 95%; 91 min,<br>solvent A 95%; 98 min

Table S4. The equipment and operation condition of HPLC analysis for carotenoids

Equipment	Agilent 1100 series (Agilent, Massy, France)
Column	YMC S-3μm column (250 × 4.6 mm; YMC Co., Kyoto, Japan)
Oven Temperature	40 °C
Detector	Photodiode Array (PDA)
Detector Wavelength	450 nm
Flow Rate	1.0 mL/min
Mobile Phase	92:8 (v/v) methanol : water with 10 mM ammonium acetate, solvent A and methyl tert-butylether, solvent B
Gradients	solvent B 10%; 0 min,<br>solvent B 17%; 20 min,<br>solvent B 25%; 29 min,<br>solvent B 70%; 35 min,<br>solvent B 70%; 40 min,<br>solvent B 75%; 42 min,<br>solvent B 10%; 45 min,<br>solvent B 10%; 55 min

Table S5. The equipment and operation condition of HPLC analysis for glucosinolates

Equipment	Agilent Technologies 1200 series HPLC system (Palo Alto, CA, USA)
Column	Reversed-phase Inertsil ODS-3 column (150 × 3.0 mm, 3 μm)<br>with an E type cartridge guard column (10 × 2.0 mm, 5 μm)
Oven Temperature	40 °C
Detector	UV-Vis
Detector Wavelength	227 nm
Flow Rate	1.0 mL/min
Mobile Phase	water, solvent A and acetonitrile, solvent B
Gradients	solvent B 7%; 0 min,<br>solvent B 24%; 18 min,<br>solvent B 24%; 32 min,<br>solvent B 7%; 40 min

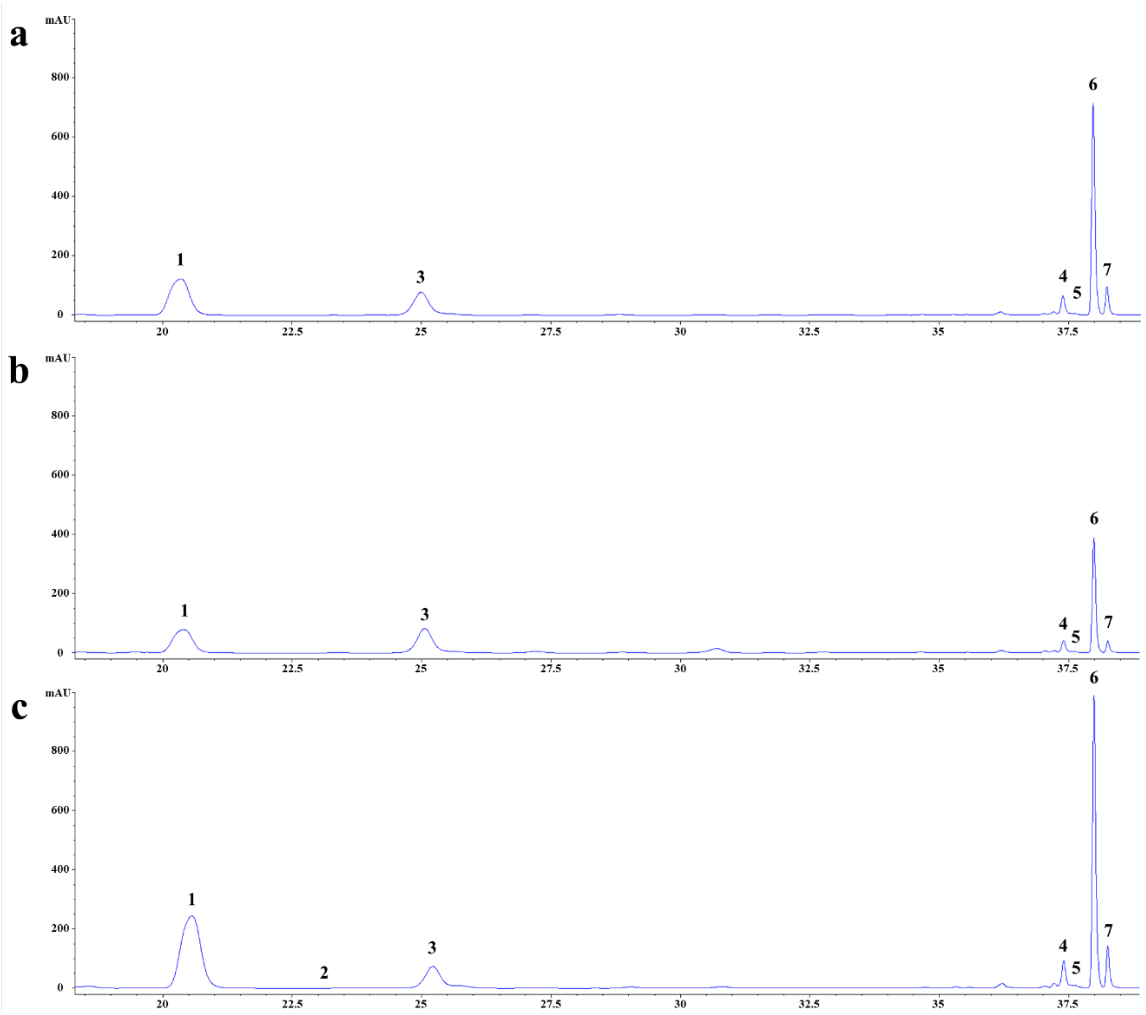

Figure S1. HPLC chromatograms of carotenoids extracted from Pakchoi (a), Tatsoi (b), and Dacheongchae (c). Peak: 1, Lutein; 2, Zeaxanthin; 3, trans-β-Apo-8`-carotenal; 4, 13Z-β-carotene; 5, α-Carotene; 6, trans-β-Carotene; 7, 9Z-β-carotene.

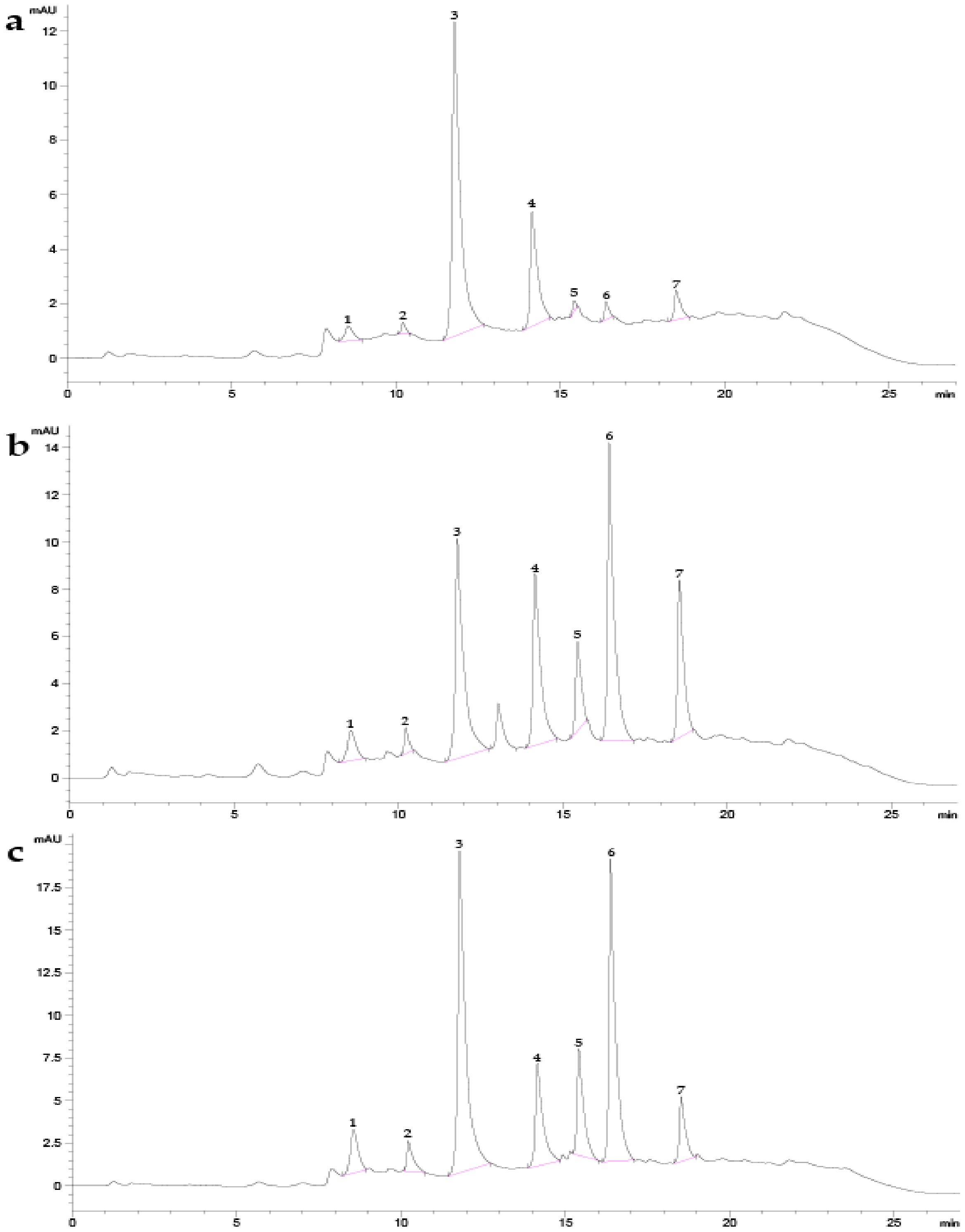

Figure S2. HPLC chromatograms of glucosinolates extracted from Tatsoi (a), Dacheongchae (b), and Pakchoi (c). Peak: 1, Progoitrin; 2, Glucoalyssin; 3, Gluconapin; 4, Glucobrassicanapin; 5, Glucobrassicin; 6, 4-Methoxyglucobrassicin; 7, Neoglucobrassicin.

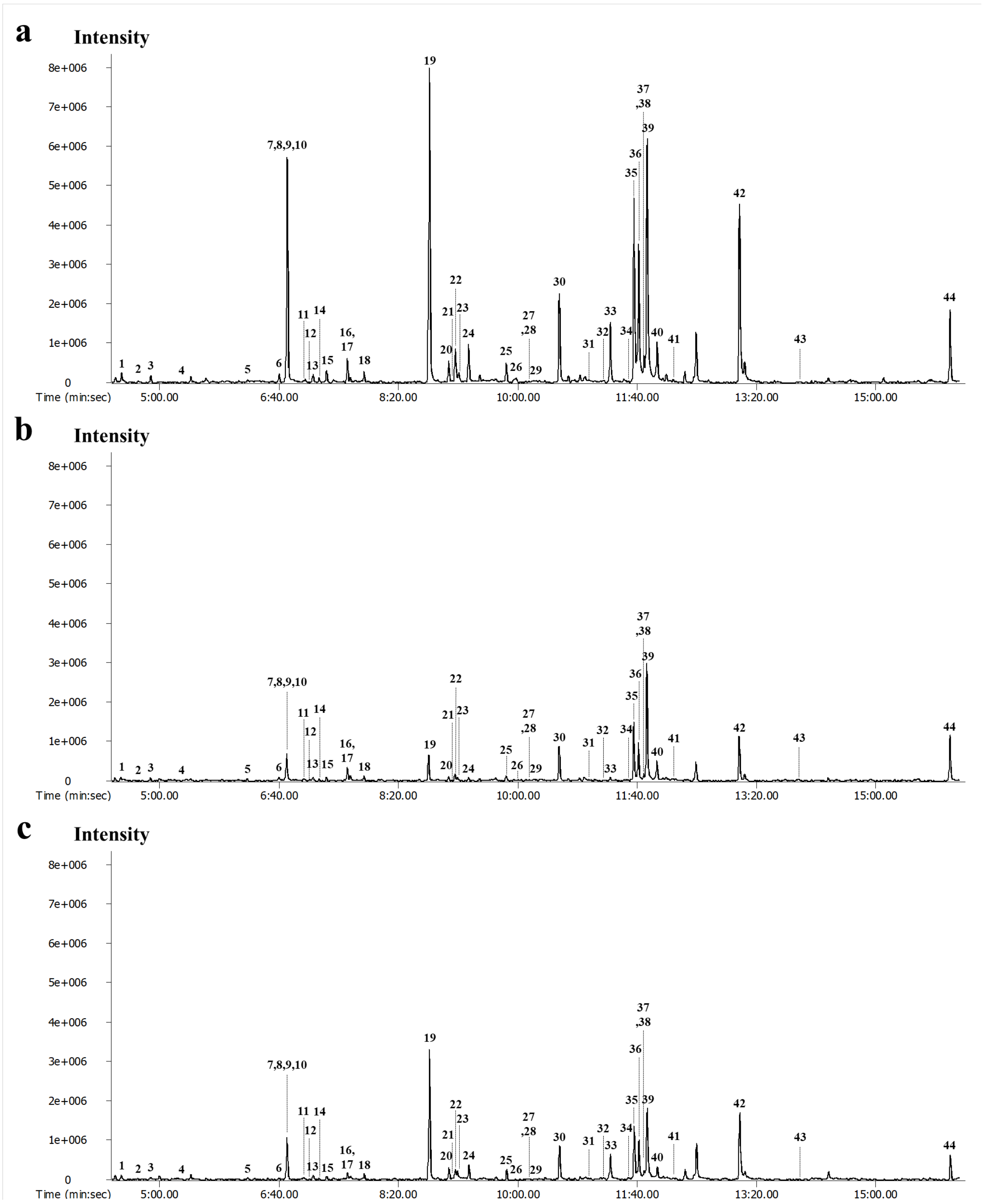

Figure S3. GC-TOF-MS chromatograms of hydrophilic metabolites obtained from Pakchoi (a), Tatsoi (b), and Dacheongchae (c). Peak: 1, Lactic acid; 2, Glycolic acid; 3, Alanine; 4, Oxalic acid; 5, Valine; 6, Serine 1; 7, Ethanolamine; 8, Phosphoric acid; 9, Glycerol; 10, Leucine; 11, Isoleucine; 12, Proline; 13, Glycine; 14, Succinic acid; 15, Glyceric acid; 16, Fumaric acid; 17, Serine 2; 18, Threonine; 19, Malic acid; 20, Aspartic acid; 21, Methionine; 22, Pyroglutamic acid; 23, 4-Aminobutanoic acid; 24, Threonic acid; 25, Glutamic acid; 26, Phenylalanine; 27, Xylose; 28, Arabinose; 29, Asparagine; 30, Ribitol; 31, Glutamine; 32, Shikimic acid; 33, Citric acid; 34, Quinic acid; 35, Fructose 1; 36, Fructose 2; 37, Mannose; 38, Galactose; 39, Glucose 1; 40, Glucose 2; 41, Tyrosine; 42, Inositol; 43, Tryptophan; 44, Sucrose.

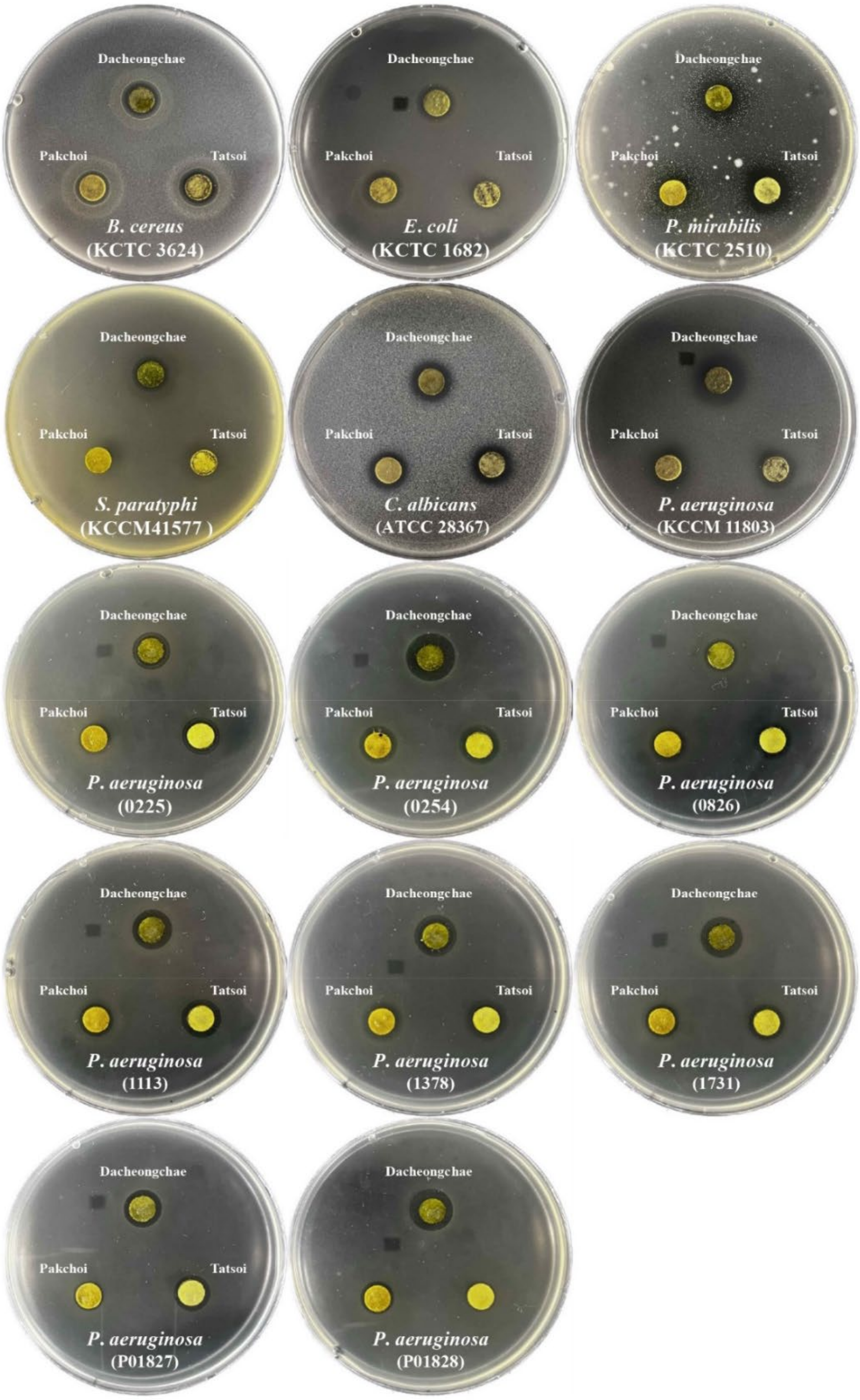

Figure S4. Representative images showing antibacterial activities of methanol extracts of Tatsoi, Dacheongchae, and Pakchoi. (right, extracts of Tatsoi ; middle, extracts of Dacheongchae; left, extracts of Pakchoi).
